# Targeted Proteomics upon Treatment with Tofersen Identifies Novel Response Markers for Superoxide Dismutase 1‐Linked Amyotrophic Lateral Sclerosis

**DOI:** 10.1002/ana.70025

**Published:** 2025-08-09

**Authors:** Christina Steffke, Karthik Baskar, Franziska Bachhuber, Maximilian Wiesenfarth, Johannes Dorst, Joachim Schuster, Florian Schöberl, Peter Reilich, Martin Regensburger, Alexander German, René Günther, Maximilian Vidovic, Susanne Petri, Thomas Meyer, Tim Hagenacker, Julian Grosskreutz, Ute Weyen, Patrick Weydt, Thomas Haarmeier, Paul Lingor, Joachim Wolf, Andreas Hermann, Johannes Prudlo, Kornelia Günther, Antje Knehr, Zeynep Elmas, Zeljko Uzelac, Simon Witzel, Wolfgang Philipp Ruf, Axel Freischmidt, Ritchie Ho, Albert C. Ludolph, Jochen H. Weishaupt, Hayrettin Tumani, Patrick Oeckl, David Brenner, Alberto Catanese

**Affiliations:** ^1^ Department of Neurology Ulm University Ulm Germany; ^2^ Institute of Anatomy and Cell Biology Ulm Germany; ^3^ International Graduate School Ulm University Ulm Germany; ^4^ German Center for Neurodegenerative Diseases (DZNE) Ulm Ulm Germany; ^5^ Department of Neurology with Friedrich Baur Institute LMU University Hospital München Germany; ^6^ Department of Molecular Neurology Friedrich‐Alexander‐University Erlangen‐Nürnberg (FAU) Erlangen Germany; ^7^ Department of Neurology University Hospital Carl Gustav Carus, Technical University of Dresden Dresden Germany; ^8^ German Center for Neurodegenerative Diseases (DZNE) Site Dresden Dresden Germany; ^9^ Department of Neurology Hannover Medical School Hannover Germany; ^10^ Department of Neurology, Center for ALS and Other Motor Neuron Disorders, Charité – Universitätsmedizin Berlin, Corporate Member of Freie Universität Berlin Humboldt‐Universität zu Berlin, and Berlin Institute of Health Berlin Germany; ^11^ Department of Neurology and Center for Translational Neuro and Behavioral Sciences (C‐TNBS) University Hospital Essen Essen Germany; ^12^ Precision Neurology of Neuromuscular and Motoneuron Diseases University of Lübeck Lübeck Germany; ^13^ Department of Neurology Ruhr‐University Bochum, BG‐Kliniken Bergmannsheil Bochum Germany; ^14^ Department for Neuromuscular Diseases Bonn University Bonn Germany; ^15^ German Center for Neurodegenerative Diseases (DZNE) Site Bonn Bonn Germany; ^16^ Department of Neurology Helios Klinikum Krefeld Krefeld Germany; ^17^ Department of Neurology TUM Klinikum Rechts der Isar München Germany; ^18^ German Center for Neurodegenerative Diseases (DZNE) Site Munich Munich Germany; ^19^ Munich Cluster for Systems Neurology (SyNergy) Munich Germany; ^20^ Department of Neurology, Diako Brüderklinikum Julia Lanz Mannheim Germany; ^21^ Translational Neurodegeneration Section ‘Albrecht Kossel’ University Medical Center Rostock Rostock Germany; ^22^ German Center for Neurodegenerative Diseases (DZNE) Rostock/Greifswald Germany; ^23^ Department of Neurology University Medical Center Rostock Rostock Germany; ^24^ Center for Neural Science and Medicine Cedars‐Sinai Medical Center Los Angeles CA USA; ^25^ Board of Governors Regenerative Medicine Institute Cedars‐Sinai Medical Center Los Angeles CA USA; ^26^ Department of Biomedical Sciences Cedars‐Sinai Medical Center Los Angeles CA USA; ^27^ Department of Neurology Cedars‐Sinai Medical Center Los Angeles CA USA; ^28^ Division for Neurodegenerative Diseases, Neurology Department, Mannheim Center for Translational Medicine University Medicine Mannheim, Heidelberg University Mannheim Germany; ^29^ Center for Rare Diseases (ZSE) Ulm Ulm University Hospital Center for Rare Diseases Ulm Germany; ^30^ Institute of Anatomy and Cell Biology, Department of Neuroanatomy Freiburg University Freiburg Germany

## Abstract

**Objective:**

Tofersen is the first effective and approved therapy for superoxide dismutase 1 (*SOD1*)‐associated amyotrophic lateral sclerosis (ALS [SOD1‐ALS]). Following treatment with tofersen, neurofilament levels in patients' cerebrospinal fluid (CSF) and serum seem to respond earlier than clinical parameters. This evidence prompted us to hypothesize that this novel treatment could provide an opportunity to identify additional biomarkers responsive to therapy in SOD1‐ALS.

**Methods:**

We investigated a panel of 120 neural, glial, and inflammatory markers in CSF and serum samples longitudinally collected from a total of 28 SOD1‐ALS patients at baseline, and after 3, 6 and 12 months of treatment with tofersen, followed by validation with conventional methodology.

**Results:**

We identified a set of proteins, including neurofilament light chain, neurofilament heavy chain, amyloid‐beta 1–40 and amyloid‐beta 1–42, neuropeptide Y (NPY), and ubiquitin C‐terminal hydrolase L1 (UCHL1), whose CSF levels both differed between SOD1‐ALS and the control group, and were responsive to tofersen at 3 and 6 months after treatment initiation. Another group of markers, including the neuropentraxin (NPTX) family members NPTX1, NPTX2 and NPTXR, did not separate untreated SOD1‐ALS from controls, but was responsive to tofersen. At 12 months on tofersen the levels of neurofilament light chain, neurofilament heavy chain, NPTX1, NPTX2, and NPTXR remained reduced compared with baseline, and correlated with the clinical response to tofersen. Consistent with increasing CSF pleocytosis and intrathecal immunoglobulin production, inflammatory markers were significantly increased after 12 months of treatment.

**Interpretation:**

Our results highlight a complex, time‐dependent differential response of CSF biomarkers to tofersen treatment, and may pave the way for developing a panel of responsive proteins to make biomarker endpoints more robust in clinical trials for SOD1‐ALS and beyond. ANN NEUROL 2025;98:1318–1334

The VALOR trial and its open label extension demonstrated the efficacy of the antisense oligonucleotide (ASO), tofersen, in familial amyotrophic lateral sclerosis (ALS) caused by variants in the *SOD1* gene (SOD1‐ALS). Additionally, they also highlighted the potential of neurofilaments as surrogate and prediction markers for the clinical response to an antineurodegenerative therapy.^1^ The levels of the axonal marker neurofilament light chain (NfL) in serum and cerebrospinal fluid (CSF) showed a treatment response earlier than clinical functional scores, what has recently been replicated in several real‐world cohorts of SOD1‐ALS patients treated with tofersen under the early access program (EAP) and since its approval.^2^, ^3^, ^4^, ^5^ This observation encourages the use of neurofilaments as the primary endpoint for future (exploratory) clinical proof‐of‐concept trials in ALS.^6^, ^7^, ^8^ Indeed, the NfL dynamics upon treatment with a candidate therapy has influenced go/no‐go decisions of recent clinical trials.^9^ In the NCT03626012 trial testing BIIB078, an antisense oligonucleotide targeting C9orf72 RNA sense strand, the patients who received the highest dose tended to show a faster disease progression and an increase in plasma and CSF NfL levels.^9^ This observation suggests that NfL dynamics may also inform clinical worsening upon an experimental therapy in clinical trials. However, as neurofilament levels can be potentially influenced by a candidate drug through other means than slowing down neurodegeneration (eg, by impacting post‐translational modifications, turnover, or expression), complementary response markers could be useful to make biomarker endpoints more robust.

We hypothesized that tofersen, as a drug with a large effect size, would offer the opportunity to assess the therapy‐responsiveness of other protein markers, as for serum troponin T.^10^ To this end, we leveraged the highly sensitive NUcleic acid Linked Immuno‐Sandwich Assay (NULISA™),^11^ to analyze a predefined panel of 120 protein targets in serum and CSF samples of 9 SOD1‐ALS patients treated with tofersen and 9 matched control subjects. The NULISA™ technology has already proved its added value in multiple neurodegenerative diseases,^12^, ^13^, ^14^ as it improves the sensitivity of traditional proximity ligation assays to attomolar level and suppresses assay background.^10^ This approach confirmed neurofilament heavy chain (NfH) and NfL as the best performing diagnostic and therapeutic markers, and identified a panel of other CSF proteins, including ubiquitin C‐terminal hydrolase L1 (UCHL1), amyloid‐beta (Aβ)1–40, Aβ1–42, and the neuropentraxin (NPTX) family members, as early response markers.

## Methods

### Participants

Patients were recruited and enrolled from the MND‐NET, a clinical and scientific network of 25 German motor neuron disease centers. We continuously collected data from 28 ALS patients with *SOD1* mutations who participated in the German tofersen early access program (EAP) and after approval of tofersen continued being followed up.^5^ All patients were diagnosed with definite, probable, or laboratory‐supported ALS according to the revised El Escorial criteria, and were positively tested for a pathogenic *SOD1* variant. The inclusion criteria for participation in the EAP comprised the patients' informed consent. Apart from riluzole, recruited patients did not receive any further disease‐modifying drug therapy. Patients participating in the long‐term extension of the placebo‐controlled phase III tofersen study (VALOR) were excluded from the EAP and this study. The EAP included a dosing phase with intrathecal administration of 100 mg tofersen at day 1, 14, and 28, and a subsequent maintenance phase during which patients received doses at intervals of approximately 28 days (at least 21 days). The cohort contains clinical data of 28 patients, which have been published previously by Wiesenfarth et al.^5^

For the NULISA™ discovery analysis, 9 of these SOD1‐ALS patients were selected according to the most pronounced reduction of CSF/serum neurofilament levels after 3 months of treatment with tofersen. Individuals of the healthy control group suffered from non‐neurodegenerative conditions, including tension headache, idiopathic intracranial hypertension, and idiopathic facial nerve paralysis.

For validation of neurofilaments, UCHL1, and Aβ‐proteins, CSF samples collected from a total of 22 patients treated with tofersen were used, as for 1 patient in the discovery cohort, there were no CSF samples for the 6‐month timepoint available.

To explore long‐term therapy‐responsive biomarkers after 12 months of tofersen treatment, CSF samples collected from 26 SOD1‐ALS patients at baseline (BL) and a time range between 11 and 13 months after treatment initiation were analyzed.

The CSF samples from the individuals recruited in the control group were collected once and not longitudinally.

Serum was obtained from peripheral blood after centrifugation (2000 *g*, 10 min), and CSF was collected by lumbar puncture. Afterwards, all samples were stored within 2 h at −80°C until further analysis.

### NULISAseq Assay

The NULISAseq CNS Disease Panel 120 allows highly sensitive and specific multiplexed analysis of 120 neurospecific and inflammatory response proteins at the same time. This panel consists of established biomarkers for various neurodegenerative diseases or candidate markers. Many of these proteins have previously not been measured or, due to methodological limitations, could not be measured in serum or CSF, respectively. NULISAseq assays were performed at Alamar Biosciences (Fremont, CA, USA), as described previously.^11^ Briefly, serum and CSF samples stored at −80°C were thawed on ice and centrifuged at 10,000 *g* for 10 mins. Then, 10‐uL supernatant samples were plated in 96‐well plates and analyzed with Alamar's CNS Disease Panel targeting mostly neurodegenerative disease‐related targets, as well as inflammation and immune response‐related cytokines and chemokines. A Hamilton‐based automation instrument was used to perform the NULISAseq workflow, starting with immunocomplex formation with DNA‐barcoded capture and detection antibodies, followed by capturing and washing the immunocomplexes on paramagnetic oligo‐dT beads, then releasing the immunocomplexes into a low‐salt buffer, which were captured and washed on streptavidin beads. Finally, the proximal ends of the DNA strands on each immunocomplex were ligated to generate a DNA reporter molecule containing both target‐specific and sample‐specific barcodes. DNA reporter molecules were pooled and amplified by polymerase chain reaction, purified and sequenced on Illumina NextSeq 2000 (San Diego, CA, USA). The proteins that passed the quality control were identified based on the target detectability, which is the percentage of samples that are above the limit of detection for a determined target.^11^

### Biomarker Validation

Phosphorylated NfH (pNfH) was measured by sandwich enzyme‐linked immunosorbent assay (ELISA) using the phosphorylated Neurofilament H Human ELISA kit (BioVendor, R&D Systems, Brno, Czech Republic). The commercially available Simple Plex Human NF‐L Catridge for the ELLA™ microfluidic system (Bio‐Techne, Minneapolis, MN, USA) was used to measure NfL concentrations in CSF.

Fully automated chemiluminescent enzyme immunoassay (CLEIA) was applied to measure Aβ1‐40 and Aβ1‐42 via Lumipulse® G β‐Amyloid 1–40 Immunoreaction Cartridges and Lumipulse® G β‐Amyloid 1–42 Immunoreaction Cartridges, respectively (Fujirebio, Ghent, Belgium).

UCHL1 levels in CSF were measured using the Human UCH‐L1/PGP9.5 DuoSet ELISA (R&D Systems, Minneapolis, MN, USA) according to the manufacturer's instructions. CSF samples were measured in singlicates in a single run. CSF QC samples with low and high UCHL1 levels were included to monitor assay performance (intra‐assay coefficient of variation 0.3–7.8%).

### Amyotrophic Lateral Sclerosis Functional Rating Scale‐Revised Analysis and Correlation with Biomarkers

The difference in levels of biomarkers between 12 months of treatment with tofersen and BL was correlated with the delta between the observed and the expected (computed) Amyotrophic Lateral Sclerosis Functional Rating Scale‐Revised (ALSFRS‐R) score at 12 months of tofersen treatment. The expected ALSFRS‐R score, defined as the extrapolated ALSFRS‐R score if the patient had not received tofersen treatment, was calculated according to Smith et al.^15^: ALSFRS‐R score at BL – (ALSFRS‐R slope at BL × 12 months). For the respective monthly ALSFRS‐R slope pretreatment at BL, the following formula was used: (48 – ALSFRS‐R score at BL) / disease duration in months since symptom onset. The observed ALSFRS‐R score was documented during the regular patient visits.

## Statistical Analysis

Sequencing data were processed using the NULISAseq algorithm (Alamar Biosciences). The sample‐ (SMI) and target‐specific (TMI) barcodes were quantified, and up to 2 mismatching bases or 1 indel and 1 mismatch were allowed. Intraplate normalization was performed by dividing the target counts for each sample well by that well's internal control counts. Interplate normalization was then performed using interplate control normalization, wherein counts were divided by target‐specific medians of the 3 interplate control wells on that plate. Data were then rescaled, add 1 and log2 transformed to obtain NULISA Protein Quantification units for downstream statistical analysis.

The transformed datasets were imported into Python (version 4.0.11; JupyterLab, San Diego, CA, USA) using the pandas package. A Venn diagram was constructed to visualize the commonly expressed proteins in the serum and CSF using the Matplotlib package. A heatmap with hierarchical clustering based on z‐scores was generated for all proteins in the serum and CSF using Seaborn and Matplotlib packages. Pearson's correlation was performed for the control, baseline, and tofersen groups separately using the NumPy package, along with further visualisation using heatmaps with hierarchical clustering using the Seaborn and Matplotlib packages. Bar graphs comparing the mean Pearson's correlation coefficients of the healthy controls, baseline, and tofersen were generated using GraphPad Prism 10 (San Diego, CA, USA). The 2‐D and 3‐D principal component analyses, along with k‐means clustering, were performed using the NumPy, Seaborn, Sklearn, and Matplotlib packages for all proteins in serum, all proteins in CSF, and commonly expressed proteins in serum and CSF. Principal component loadings were extracted from the principal component analysis by creating a new data file using the pandas package and plotted as bar graphs using the matplotlib package. Pearson's correlation was performed between biomarkers. Scatterplots to visualize correlations were generated in Python using the seaborn package.

Microsoft Excel and GraphPad Prism (version 10) were used for data collection and statistical analysis with the following statistical tests: in case of normally distributed data, two independent groups were compared using the unpaired *t* test with Welch's correction, in case of non‐normal distribution, nonparametric Mann–Whitney test was applied. Tofersen and baseline condition were analyzed using the paired *t* test for normally distributed data, and Wilcoxon matched‐pairs signed rank test in case of non‐normal distribution comparing samples from similar patients. To evaluate differences among multiple groups, 1‐way ANOVA followed by the post‐hoc Tukey test was used. Statistical significance was set at *p* < 0.05. All data analyzed by paired *t* test are shown as the mean, including comparison lines for paired data points (patients).

## Graphical Design

Graphical illustrations shown in the figures were adapted from images provided by Servier Medical Art (Servier; https://smart.servier.com/) licensed under a Creative Commons Attribution 4.0 Unported License.

## Ethics

Biosampling of serum and CSF was conducted via participation in the MND‐NET cohort study, for which informed consent was obtained. The MND‐NET cohort study was approved by the ethics committee of Ulm University (application number: 19/12).

## Results

The highly effective antineurodegenerative tofersen treatment represents a unique opportunity to identify novel therapy‐responsive biomarkers for SOD1‐ALS. Thus, we formed a discovery cohort (of responder patients) by selecting the 9 SOD1‐ALS individuals from the group of patients previously recruited in the German EAP who showed the most pronounced reduction of CSF and serum neurofilament upon tofersen therapy (Table S1).^5^ The healthy control group consisted of patients with non‐neurodegenerative neurological conditions, including tension headache, idiopathic intracranial hypertension, and idiopathic facial nerve paralysis (Table S2).

**Fig. 1:**
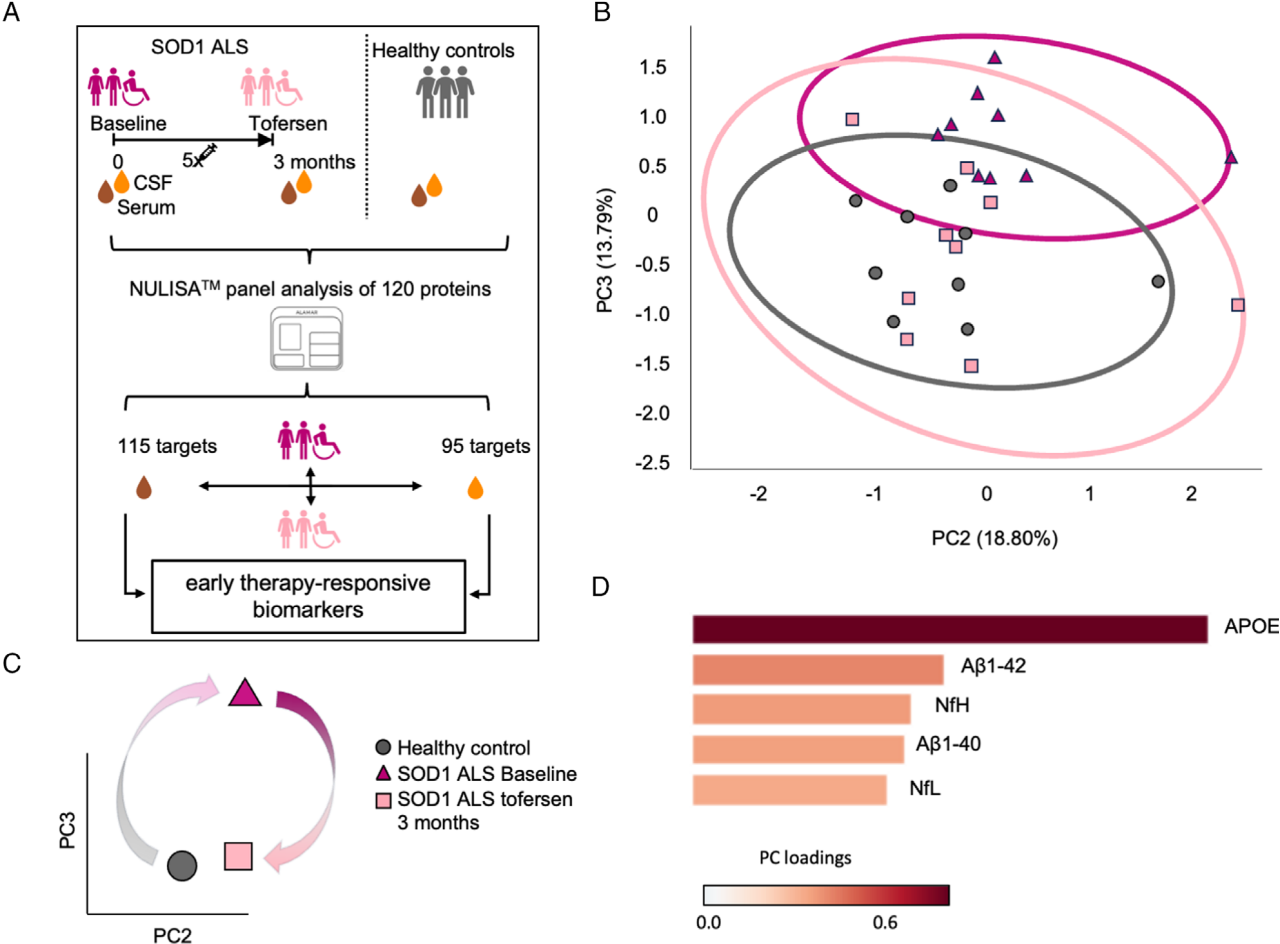
Cerebrospinal fluid (CSF) shows a proteomic fingerprint indicative of neuroprotection after 3 months of treatment with tofersen. (A) Schematic representation of the study design for the initial cohort: longitudinal collection of CSF and serum sample pairs from superoxide dismutase 1 (*SOD1*)‐associated amyotrophic lateral sclerosis (SOD1‐ALS) patients was performed at baseline and at 3 months after tofersen treatment initiation, as well as from age‐ and sex‐matched controls with minor neurological conditions. Both biofluids were then analyzed with a NULISA™ panel platform including 120 proteins. (B) Principle components (PC)2 and PC3 show a clear transition from tofersen‐treated patients close to the healthy controls, underlined by (C) centroid representation of PC2 and PC3. (D) Apolipoprotein E4 (APOE), amyloid beta (Aβ)1–42, neurofilament heavy chain (NfH), Aβ1‐40 and neurofilament light chain (NfL) have the highest load in of PC2 and PC3 combined. [Color figure can be viewed at www.annalsofneurology.org]

We used the ultrasensitive NULISA™ array to investigate a panel of 120 neural, glial, and inflammatory markers in CSF and serum sample pairs longitudinally collected from the discovery cohort at baseline and 3 months after the beginning of tofersen treatment (Fig. 1A). In the discovery phase of our biomarker analysis, we focused on the early 3 months timepoint to highlight immediate effects at the proteome level and minimize effects linked to disease progression.

We identified 115 proteins for serum and 95 for CSF samples that passed quality control and were selected for further analysis (Fig. 1A). Unbiased hierarchical clustering performed with the 91 proteins commonly detected in both biofluids showed a clear separation between the CSF and serum proteome, but could not cluster the samples according to the 3 groups of individuals (healthy controls, SOD1‐ALS baseline, and SOD1‐ALS tofersen; Fig. S1A–C). Accordingly, patient‐centric correlation analysis highlighted a higher degree of heterogeneity within the serum and CSF targeted proteome of the SOD1‐ALS patients at baseline than in the healthy controls. A higher similarity was observed in CSF, whereas serum samples showed the highest interpatient heterogeneity, which was partially normalized upon tofersen treatment (Fig. S1D,E). Thus, we proceeded to analyze both biofluids separately.

We first focused on serum, in which the SOD1‐ALS samples presented the highest variability. By comparing the protein panel of SOD1‐ALS patients at baseline to controls, we identified NfL and the Tau isoforms, pTau‐231, pTau‐181, and total Tau, as significantly upregulated after correction for multiple comparisons. When looking at the unadjusted *p* value, 26 molecules were significantly altered in the ALS group: interleukin (IL)‐15, IL‐16, and Aβ1‐40 were downregulated, whereas the levels of the aforementioned markers, other interleukins, and several cytokines were higher in disease samples (Figs S2 and S3A,B). This confirmed that the NULISA™ technology can identify a SOD1‐ALS fingerprint in serum. Nevertheless, despite SOD1‐ALS samples being effectively separated from controls by principal component analysis when considering the principle components (PC) 2 and 3, the tofersen group remained close to its baseline counterparts (Fig. S4A,B). We then looked at the combined PC loadings of PC2 and PC3, which highlighted apolipoprotein E4, NfH, and NfL as the proteins mostly contributing to the (reduced) shift observed from an untreated to treated state (Fig. S4C). In agreement with these findings, paired comparison (without correction for multiple comparisons) of the untreated and treated SOD1‐ALS samples confirmed a significant reduction of NfH and NfL.^1^, ^5^ Furthermore, we detected a significant increase of glial fibrillary acidic protein (GFAP), IL‐9, secreted modular calcium‐binding protein 1, NPTX2, Aβ1–40, IL‐15, TAFA5, NPY, and vascular cell adhesion molecule 1upon tofersen treatment (Fig. S5A–C). Apart from neurofilaments, only Aβ1–40 was inversely altered in untreated SOD1‐ALS versus controls, indicating only these markers were reverted by the tofersen treatment in serum (Fig. S6).

We then investigated the effect of tofersen treatment on the CSF. First, we compared untreated SOD1‐ALS with controls, and found that the levels of NCAM1, NPTXR, GOT1, PSEN1, different Tau forms, as well as SOD1,^16^ were significantly decreased in patients compared with controls. Expectedly, the concentrations of several established ALS biomarkers, such as NfH, NfL, UCHL1, and CHIT1, were increased in patients (Figs S7 and S8A,B). According to these alterations in the CSF of SOD1‐ALS patients, PC1 could clearly separate the disease status from controls, but, when used in combination with PC2, could not distinguish between untreated and tofersen samples. In contrast, the combination of PC2–PC3 represented the best strategy to identify a neuroprotective effect driven by tofersen, as these 2 PCs showed a clear distinction between a treated and non‐treated ALS condition, with the proteome profile of the ALS patients that was shifted back in close proximity to the healthy control group upon tofersen treatment (Fig. 1B,C). This indicated that the proteins responsible for this shift might represent the fingerprint for a neuroprotective effect in SOD1‐ALS. Therefore, we aimed at identifying the panel targets underlying the effect of tofersen and looked at the PC loadings in an analogous manner as for the serum samples. We identified apolipoprotein E4, Aβ1‐42, Aβ1‐40, NfH, NfL, and NPY as the proteins with the highest significance in PC2 and PC3 (Fig. 1D).

**Fig. 2:**
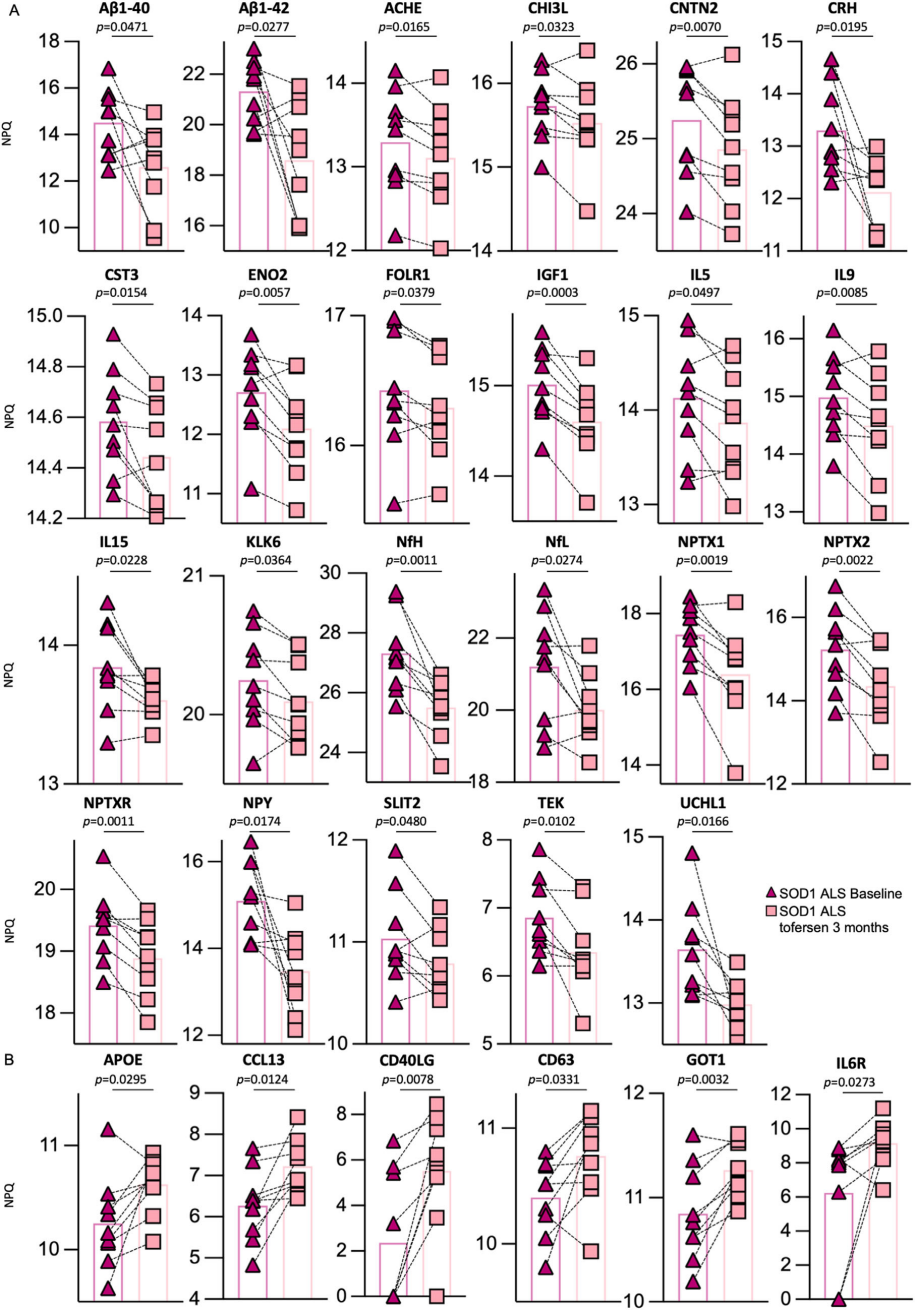
Paired analysis of significantly changed proteins according to linear mixed effects model between 3 months of treatment with tofersen and baseline condition in cerebrospinal fluid. Significantly (A) downregulated and (B) upregulated proteins in paired *t* testing. Statistical significance was set at *p* < 0.05, exact *p* values are shown. SDO1‐ALS, superoxide dismutase 1‐associated amyotrophic lateral sclerosis. [Color figure can be viewed at www.annalsofneurology.org]

We then performed a paired comparison of CSF samples from SOD1‐ALS patients at baseline and with 3 months of tofersen, revealing 23 and 6 proteins being significantly down‐ or upregulated, respectively (Fig. S9). Specifically, the levels of ACHE, Aβ1–40, Aβ1–42, CHI3L, CNTN2, CRH, CST3, ENO2, FOLR1, IGF1, IL‐5, IL‐9, IL‐15, KLK6, NfH, NfL, NPTX1, NPTX2, NPTXR, NPY, SLIT2, TEK, and UCHL1 (Fig. 2A) were reduced by the treatment, whereas apolipoprotein E4, CCL13, CD40LG, CD63, and GOT1 were increased (Fig. 2B). Of note, besides NfH and NfL, GOT1, NPY, Aβ1–42 (strong trend also for Aβ1–40), CD40LG, and UCHL1 were inversely altered in untreated SOD1‐ALS versus controls, indicating that these markers could be useful for both diagnostic and therapeutic purposes (Fig. S10).

**Fig. 3:**
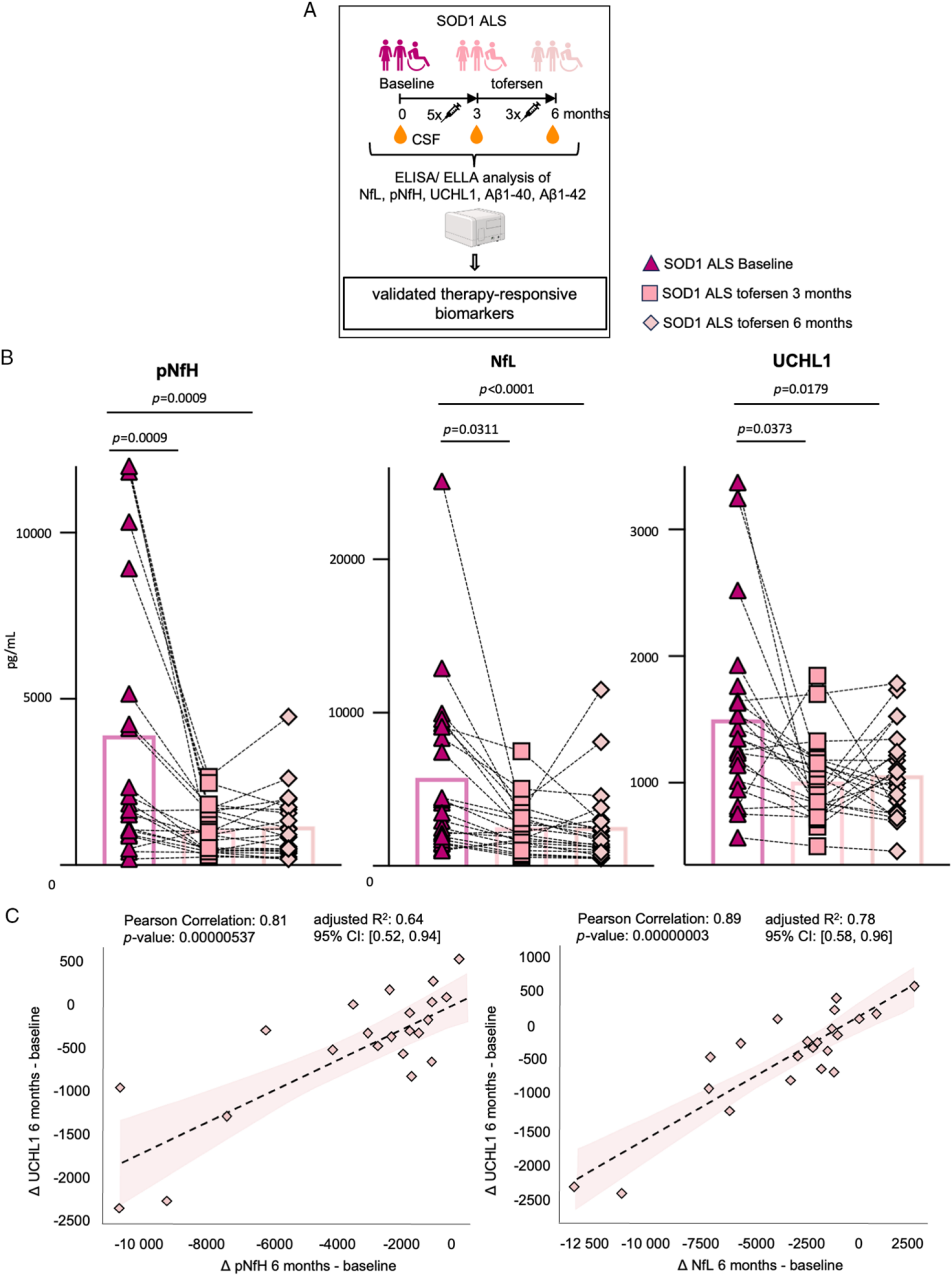
Validation of neurofilaments and UCHL1 changes upon 3‐ and 6‐month treatment with tofersen confirm results of NULISA™ panel analysis. (A) Workflow of validation experiments: cerebrospinal fluid samples of 22 tofersen‐treated patients from baseline, 3 months, and 6 months were analyzed for neurofilament heavy chain (NfH), phosphorylated NfH (pNfH), UCHL1, amyloid beta (Aβ)1–40, and Aβ1–42. (B) Measurement of neurofilament light chain (NfL), pNfH, and UCHL1 in 22 patients during a time period of baseline to 6 months of tofersen treatment shows significant downregulation after 6 months in all markers as afore indicated by the NULISA^TM^ panel analysis. (C) UCHL1 levels correlate significantly with pNfH and NfL between baseline and 6 months. Data are shown as mean value ± SD (1‐way ANOVA with post‐hoc Dunn or Tukey test). Statistical significance was set at *p* < 0.05, exact *p* values are shown. SOD1‐ALS = superoxide dismutase 1‐associated amyotrophic lateral sclerosis. [Color figure can be viewed at www.annalsofneurology.org]

Subsequently, we decided to validate if these results are also true in a clinically more heterogeneous group of SOD1‐ALS patients and with alternative methodology. Thus, we measured the CSF levels of NfL, pNfH, UCHL1, Aβ1–40, and Aβ1–42 with ELLA and ELISA at baseline, as well as 3 and 6 months after tofersen initiation in 8 of the 9 individuals of our exploratory cohort (for 1 patient, samples from the additional timepoint of 6 months were not available) plus 14 additional SOD1‐ALS patients from the previously described German trial^5^ (n = 22 patients in total; Fig. 3A). These markers were significantly altered in SOD1‐ALS patients at baseline when compared with the control group, and their levels were significantly normalized by tofersen treatment (Fig. S10). Both NfLs and neurofilament heavy chains showed a significant reduction in the CSF after 3 months of tofersen therapy compared with baseline that remained stable up to 6 months after treatment initiation, confirming the findings previously published by Wiesenfarth et al. (Fig. 3B).^5^ Equally, CSF UCHL1 levels showed a decrease after 3 months of tofersen treatment, which remained stable after another 3 months. We observed a significant correlation between the reduction of UCHL1, pNfH (Pearson's correlation = 0.81, *p* value = 0.00000537, adjusted *R* ^2^ = 0.64, 95% CI 0.52–0.94) and NfL (Pearson's correlation = 0.89, *p* value = 0.00000003, adjusted *R* ^2^ = 0.78, 95% CI 0.58–0.96; Fig. 3C).

**Fig. 4:**
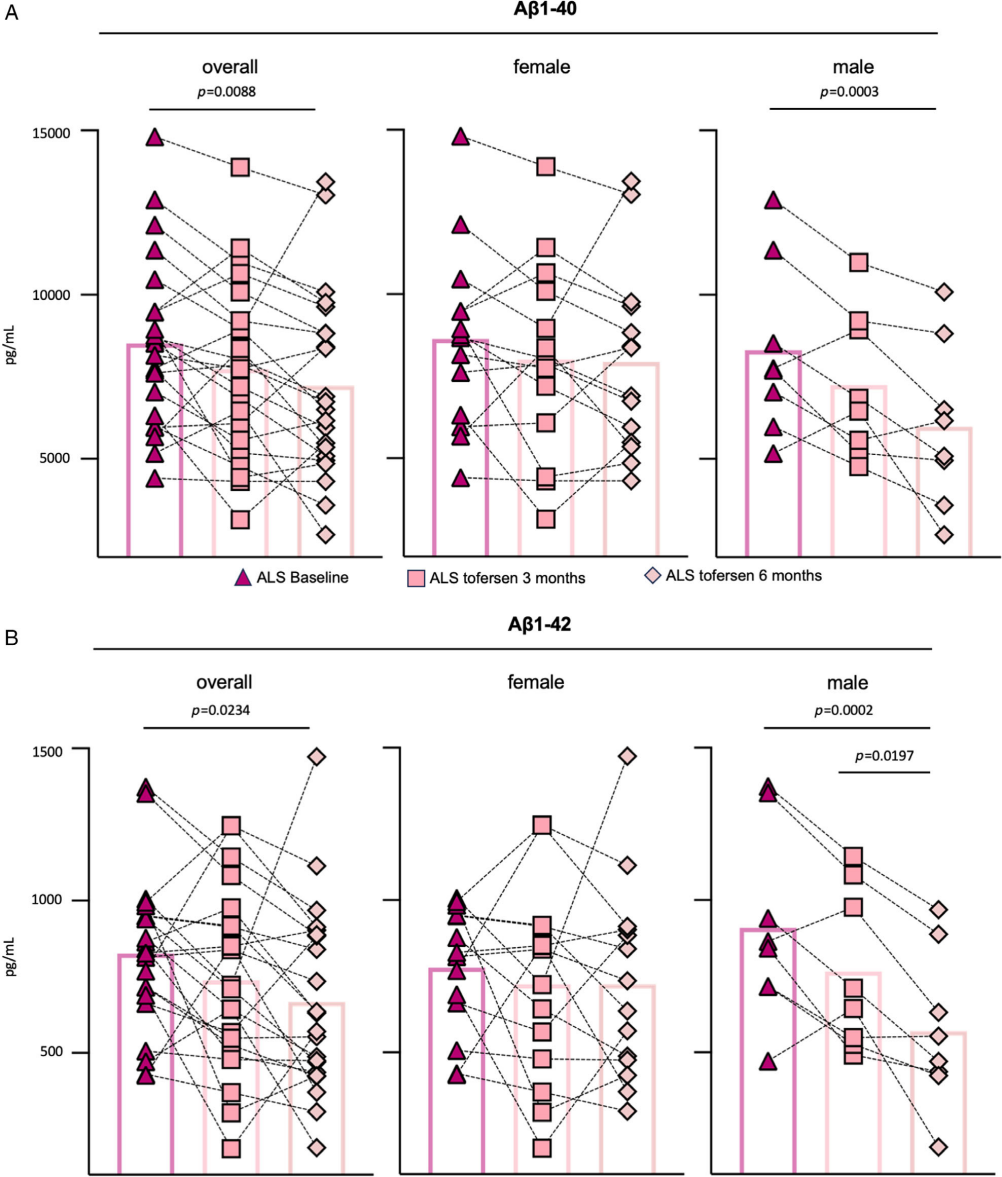
Validation of amyloid beta (Aβ) proteins at 3 and 6 months of tofersen treatment reveals a heterogeneous pattern. Aβ protein levels show a heterogeneity, yet decrease significantly after 6 months compared with baseline. Heterogeneity of (A) Aβ1–40 and (B) Aβ1–42 in the overall cohort is based on a sex dimorphism, with men (n = 8) significantly responding to tofersen treatment with a drop in both Aβ forms, whereas women (n = 14) do not show any significant dynamics. Data are shown as mean value ± SD (1‐way ANOVA with post‐hoc Dunn or Tukey test). Statistical significance was set at *p* < 0.05, exact *p* values are shown. ALS = amyotrophic lateral sclerosis. [Color figure can be viewed at www.annalsofneurology.org]

Compared with UCHL1, pNfH and NfH, both Aβ1–40 and Aβ1–42 showed a markedly higher interpatient variability. We observed a trend toward reduction of Aβ1–40 and Aβ1–42 after 3 months of tofersen treatment, which only reached statistical significance 3 months later (Fig. 4A,B). Importantly, we noticed that the high interpatient variability in Aβ‐peptide levels was sex‐dependent. Female patients (n = 14) did not show any significant change in Aβ levels upon tofersen exposure. In contrast, male patients (n = 8) presented a progressive, significant drop in the levels of both Aβ during treatment compared with baseline (Fig. 4A,B), and this behavior appeared to be specific for the Aβ peptides, as UCHL1 and the neurofilaments did not show a sex‐dependent trend (data not shown). In addition to this discrepancy, we did neither detect a significant correlation between the changes of neurofilament and Aβ peptide levels in the overall cohort, nor in the male responding patients (Fig. S11).

**Fig. 5:**
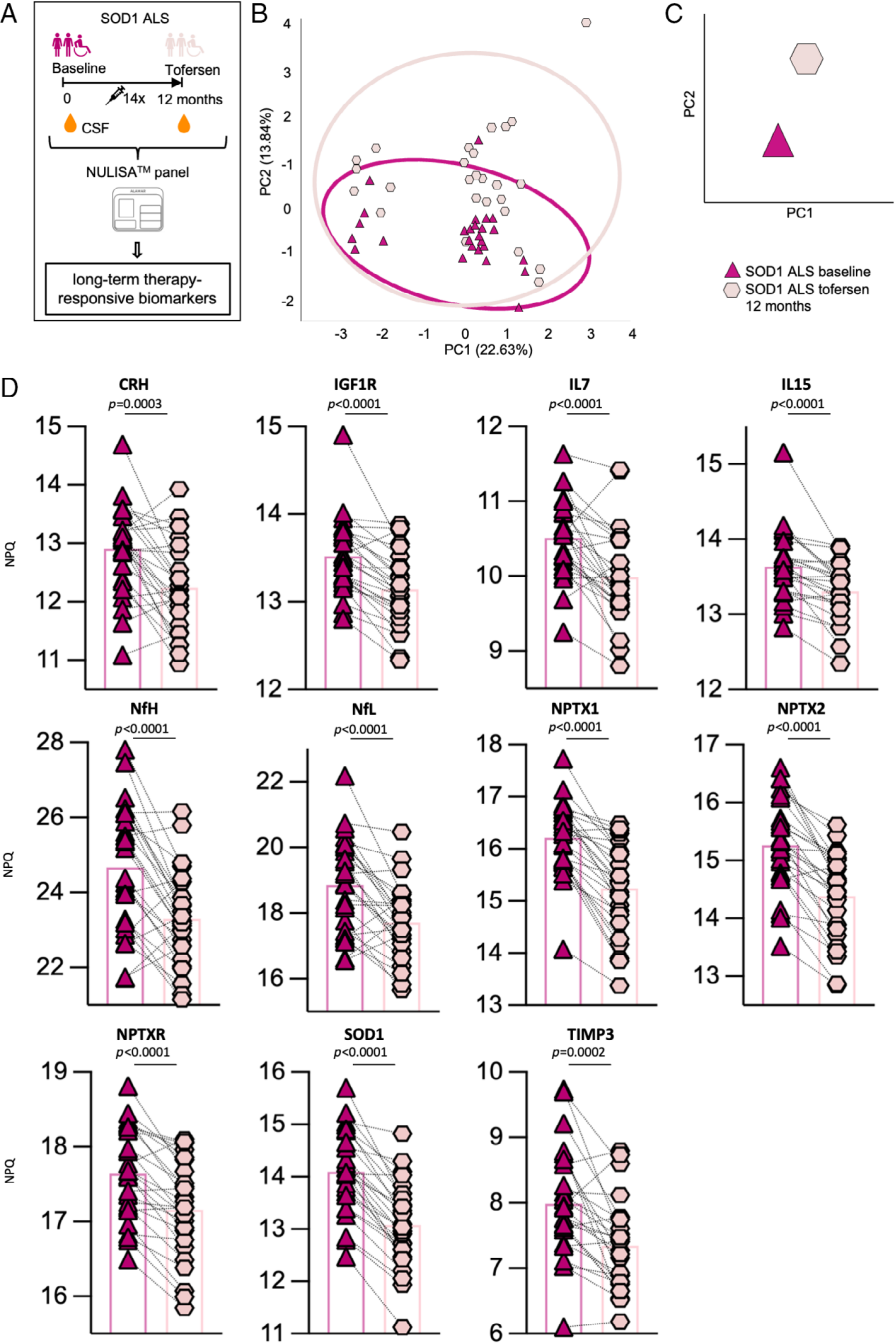
Cerebrospinal fluid (CSF) analysis after 12 months of treatment with tofersen confirms neurofilaments and highlights neuropentraxins as long‐term response markers. (A) Workflow of experiments to uncover late therapy responsive biomarkers: longitudinal collection of CSF sample pairs from superoxide dismutase 1 (*SOD1*)‐associated amyotrophic lateral sclerosis (SOD1‐ALS) patients was performed at baseline and at 12 months after tofersen treatment initiation. All biofluids were then analyzed with a NULISA™ panel platform including 120 proteins. (B) Principle components (PC) analysis shows a clear discrimination between SOD1 ALS baseline and 12 months tofersen samples in PC1 and PC2, highlighted by (C) centroid representation. (D) Significantly downregulated proteins in SOD1‐ALS patients after 12 months of tofersen treatment compared with baseline in paired *t* testing. Statistical significance was set at *p* < 0.05, exact *p* values are shown. [Color figure can be viewed at www.annalsofneurology.org]

Next, we asked if the changes observed at 3 and 6 months on tofersen would persist after a longer treatment period, and if new alterations would emerge – in particular, in view of the known pleocytosis and intrathecal antibody production, which is known to develop in the course of treatment with tofersen.^5^, ^17^ Consequently, we again made use of the NULISA panel to analyze CSF at 12 months of treatment with tofersen including the 21 patients from the validation cohort and additional 5 patients (in total n = 26; Fig. 5A). The CSF of SOD1‐ALS patients treated with tofersen for 1 year showed a clearly different proteomic profile than at earlier treatment stages (Figs 5B,C and S12). First, we found a robust upregulation of proinflammatory cytokines, interleukins, tumor necrosis factor, and GFAP, in the CSF of SOD1‐ALS patients on tofersen for 12 months compared with baseline (Fig. S13). Second, the effect of tofersen on GOT1, NPY, Aβ1–42, and UCHL1 observed at the early treatment timepoints of 3 and 6 months had disappeared at 12 months of treatment. The proteins whose levels remained altered at 12 months on tofersen compared with baseline were the neurofilaments NfL and NfH, the neuropentraxins NPTX1, NPTX2, and NPTXR, CRH, IL‐15, and SOD1 (Fig. 5D).

To exclude that this incongruence between 3 and 12 months on tofersen might have occurred due to selection bias in the discovery cohort (composed of the 9 patients with the most profound reduction of neurofilament levels in the first German cohort of tofersen‐treated SOD1‐ALS patients^5^), we specifically looked at the effect of 12 months of tofersen on these individuals. Although the neurofilament levels were still significantly downregulated at this later timepoint, we only found a trend toward reduction (*p* = 0.08) in the levels of Aβ1–42, whereas GOT1, NPY, and UCHL1 returned to baseline levels (Fig. S14A). Accordingly, unsupervised comparison of the CSF targeted proteome from the patients of the discovery cohort and the additional ones analyzed after 12 months of treatment did not highlight any clustering between the 2 subgroups of SOD1‐ALS patients (Fig. S14B). Thus, this biphasic dynamic of reactive biomarkers appears to be linked to the effect of the ASO, rather than to a patient‐specific response to the treatment.

**Fig. 6:**
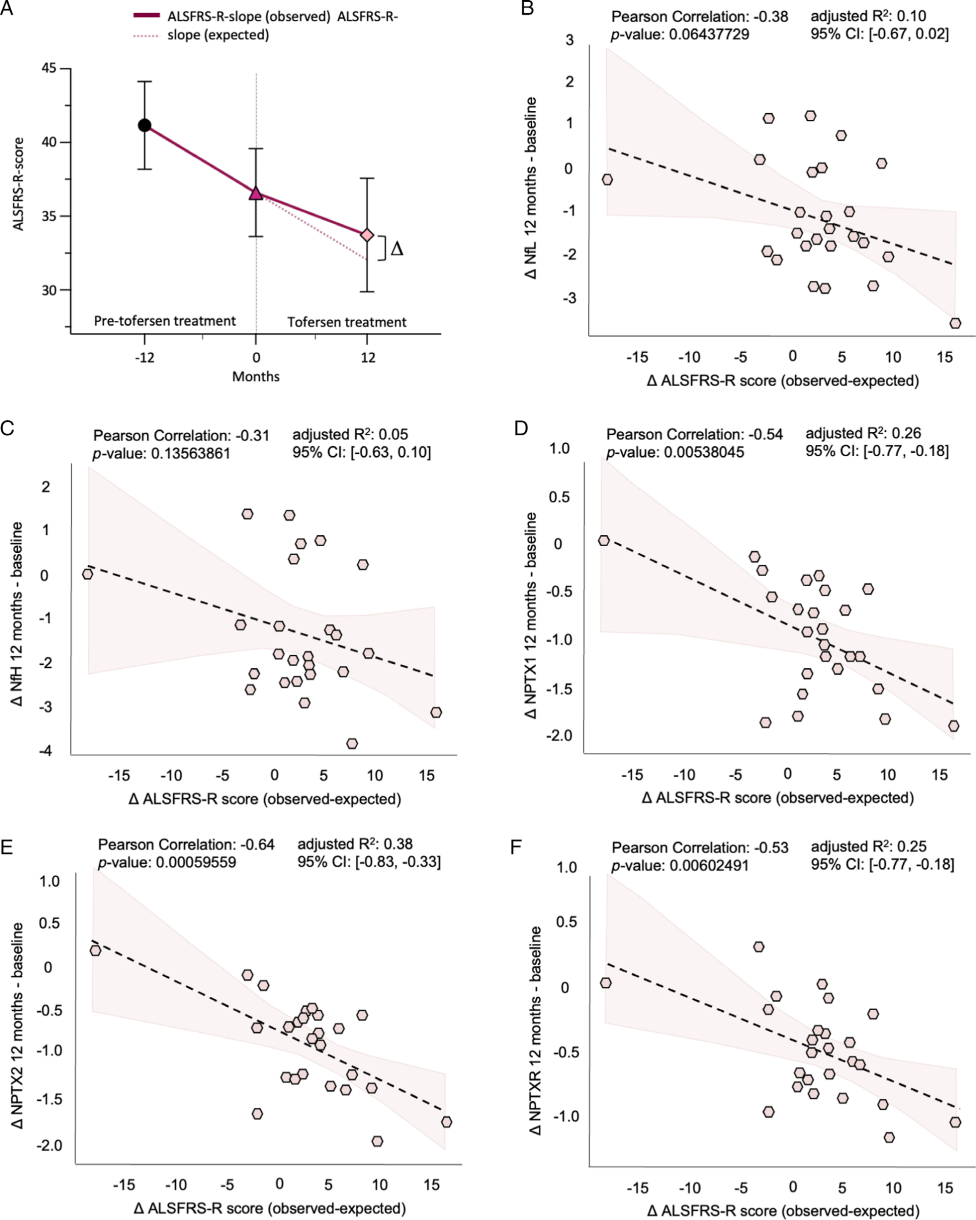
Neuropentraxins (NPTX) correlate significantly with the clinical state of superoxide dismutase 1 (*SOD1*)‐associated amyotrophic lateral sclerosis (SOD1‐ALS) patients 12 months after tofersen treatment initiation. (A) Schematic representation of the calculation of the delta in clinical progression: expected Amyotrophic Lateral Sclerosis Functional Rating Scale‐Revised (ALSFRS‐R) score was calculated as follows: ALSFRS‐R score (baseline) – individual progression rate at baseline × months on treatment. The expected ALSFRS‐R score was subtracted from the actual observed ALSFRS‐R score to show the treatment effect. Correlation analysis with the clinical outcome reveals a significant correlation with (B) NPTX1, (C) NPTX2, and (D) NPTXR. Both (E) neurofilament light chain (NfL) and (F) neurofilament heavy chain (NfH) do not significantly correlate with the clinical progress between baseline and 12 months. [Color figure can be viewed at www.annalsofneurology.org]

The persistent downregulation of IL15, CRH, and the neuropentraxin family members NPTX1, NPTX2, and NPTXR at 12 months on tofersen highlighted these proteins as promising candidate markers to monitor long‐term neuroprotection. To further evaluate their validity as long‐term response markers, we correlated the changes in protein levels after 12 months of tofersen from baseline of IL‐15, CRH, the neuropentraxins, and of the established therapy response markers NfL and NfH to the clinical response (Fig. S15). The clinical response was obtained by calculating the difference between the observed ALSFRS‐R and the expected (extrapolated) score if the patients would not have received the treatment (according to Smith et al., see Methods section and Fig. 6A).^15^ Although we found that the changes in NfL (Fig. 6B) and CRH (Fig. S16A) could be only slightly (*p* = 0.06) associated with tofersen's effects on the dynamic of the ALSFRS‐R score, IL‐15 (Fig. S16B) and NfH (Fig. 6C) did not correlate with the clinical outcome. In contrast, the reduction of NPTX1, NPTX2, and NPTXR showed a highly significant correlation with the clinical response to tofersen (NPTX1: Pearson's correlation = −0.54, *p* value = 0.00538045, adjusted *R* ^2^ = 0.26, 95% CI −0.77, −0.18; NPTX2: Pearson's correlation = −0.64, *p* value = 0.00059559, adjusted *R* ^2^ = 0.38, 95% CI −0.83, −0.33; NPTXR: Pearson's correlation = −0.53, *p* value = 0.00602491, adjusted *R* ^2^ = 0.25, 95% CI −0.77, −0.18; Fig. 6D–F).

## Discussion

This study assessed the effect of tofersen on a targeted portion of the CSF (and serum) proteome from SOD1‐ALS patients after 3, 6, and 12 months of tofersen to identify novel markers of treatment response. We opted for the multiplexed NULISA™ platform,^11^ which allowed us to test the therapy responsiveness of 120 apoptosis, neural, astrocytic, microglial, and inflammatory markers with attomolar sensitivity at the same time. The reliability of this approach was confirmed by comparing SOD1‐ALS patients before treatment with matched controls, which reproduced previously described alterations of SOD1‐ALS biomarkers including the neurofilaments NfL and NfH, SOD1, Tau‐isoforms, UCHL1, GFAP, and CHIT1.^16^, ^18^, ^19^ This analytic approach confirmed NfH and NfL as the best performing diagnostic and therapeutic biomarkers in both serum and CSF. Apart from the neurofilaments, we found a fraction of markers, namely Aβ1–42, NPY, UCHL1, and GOT1, which not only discriminated SOD1‐ALS from controls, but whose levels were also partially “corrected” by 3‐month treatment with tofersen, thus qualifying them as potential diagnostic markers and indicators of the early treatment response. However, after a treatment period of 1 year, from this group of markers only the neurofilaments NfL and NfH still showed treatment responsiveness (ie, reduction) compared with baseline. Notably, the SOD1 protein levels were reduced after 12 months on tofersen, thus strengthening the validity of the method. The well‐established ALS microglial marker, CHIT‐1, was also elevated in CSF of SOD1‐ALS patients, as previously described,^20^, ^21^ but did not show a response to tofersen treatment. Another group of markers, embracing the neuropentraxin family members NPTX1, NPTX2, and NPTXR, as well as CRH and IL‐15, did not discriminate SOD1‐ALS from the matched control group, but showed a reduction at both 3 and 12 months of therapy with tofersen, suggesting them as long‐term response markers. Yet, only the changes of the neuropentraxin levels correlated with the clinical response (Fig. 6B–D). In this context, we want to emphasize that the ALS‐FRS‐R measurements have been performed under real‐world conditions. This indicator of the clinical response was evaluated by various raters involved and in an unblinded fashion, which may have contributed to the large variation in ALS‐FRS‐R scores in some individuals during the course of treatment. In contrast, robust functional improvements have also been repeatedly reported in single individuals in other real‐world cohorts supporting our results.^15^, ^22^ For the correlation with biomarker changes, we calculated a predicted ALSFRS‐R score based on extrapolation of the progression rate at baseline before treatment start (monthly slope of ALSFRS‐R), as previously described.^15^ We are aware that these procedures are linked to at least 3 shortcomings. First, the ALSFRS‐R slope is not linear or constant/stable across the course of the disease.^23^ Second, the disease duration before the start of tofersen treatment differed between the patients. Shorter observation periods are associated with higher variation than longer ones, and the ALSFRS‐R is not sensitive for small clinical changes. Third, through extrapolation, the robustness of the ALSFRS‐R values may even have been decreased. However, in absence of a placebo group, this represents the best suitable approach to perform correlations between the biomarker and clinical changes.

Our longitudinal mapping of a selected portion of the proteome revealed some important aspects of therapy responsiveness, which might also be applied in future clinical trials. First, analysis of the CSF yielded more robust results than blood, which was in line with previous reports,^24^ and analysis of paired CSF and blood samples.^25^ These differences are likely due to the CSF composition (linked to the reduced protein content) and its close contact to the central nervous system. The differential alterations of single proteins in the 2 biofluids might be related to specific cell types or tissues, as recently shown by the selective increase in the level of muscle‐derived Tau isoforms in the blood of ALS patients.^26^ Accordingly, proteomic differences have also been documented, even when comparing CSF with the spinal cord.^19^ The biological origin of these differences still has to be explored, as it might rise from different factors, such as altered gene expression, accumulation and/or secretion of protein aggregates, or the integrity of the blood–central nervous system barrier, which is compromised in ALS.^27^

**Fig. 7:**
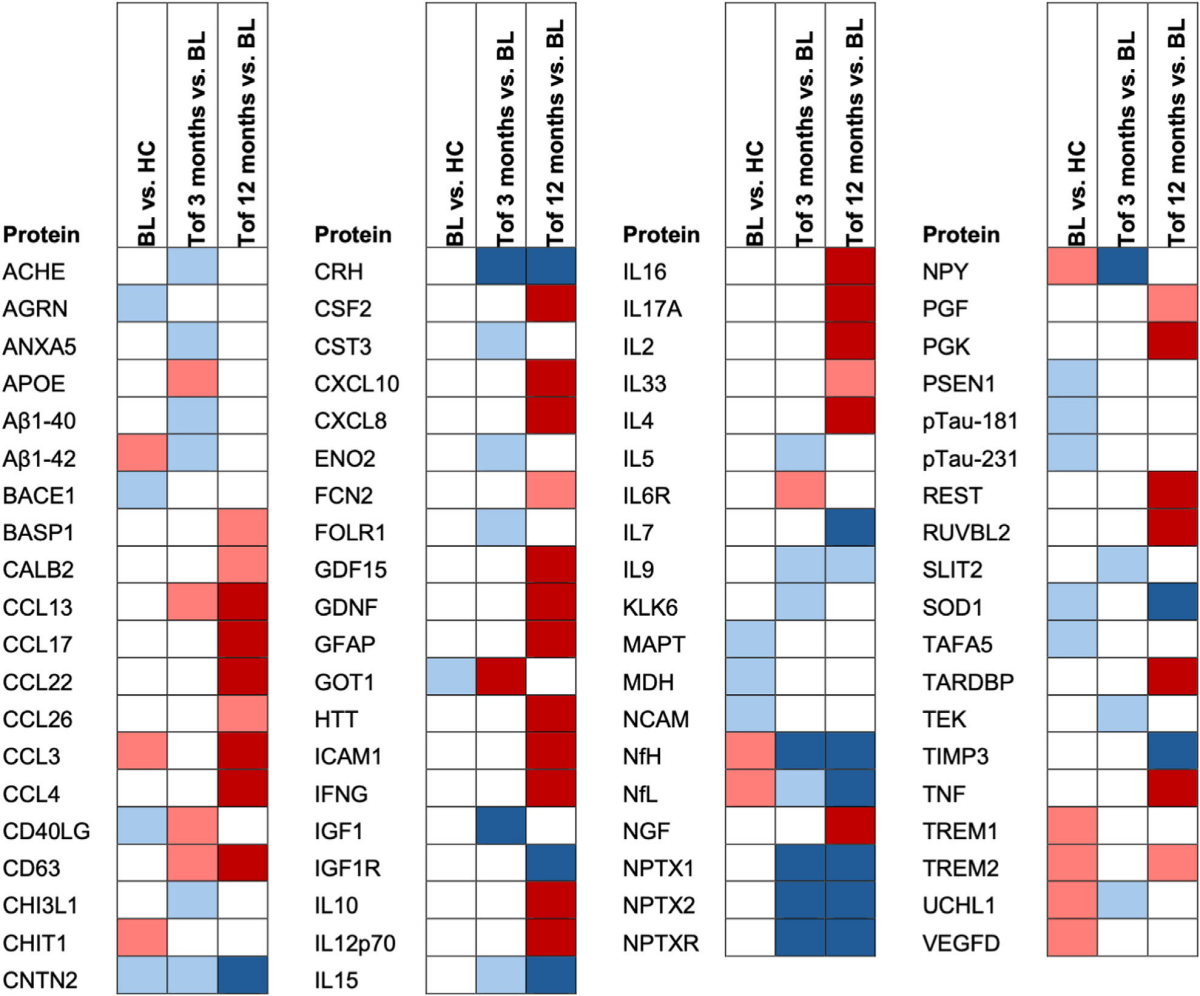
Schematic representation of the changes in the cerebrospinal fluid proteome after 3 and 12 months of tofersen treatment. Bright blue indicates significant downregulation according to unadjusted *p* value, dark blue indicates significant downregulation according to FDR‐adjusted *p* value. Bright red indicates significant upregulation according to unadjusted *p* value, dark red indicates significant upregulation according to false discovery rate (FDR)‐adjusted *p*‐value. The *p*‐value was always set as <0.05. BL = baseline; Tof = tofersen. [Color figure can be viewed at www.annalsofneurology.org]

Despite the advantage of blood collection being minimally invasive and repeatable, serum samples showed a high degree of heterogeneity, and several inflammatory and glial markers showed a heterogenous behavior in blood and CSF upon early tofersen treatment (Figs 7 and S6), reflecting differential changes in cell types and inflammatory pathways. The inflammatory response to tofersen within the CSF became much clearer after a treatment period of 12 months. In line with previous studies reporting a progressive increase of CSF pleocytosis and intrathecal immunoglobulin production during treatment,^5^, ^17^ the levels of a wide range of proinflammatory cytokines, including tumor necrosis factor and GFAP, markedly increased at 1 year treatment with tofersen. Consistently, increased CSF levels of the acute inflammatory protein serpinA1 and the glial protein CHI3L1 have been described in an independent tofersen cohort.^28^ Nevertheless, the biological meaning of such a proinflammatory response triggered by tofersen treatment still needs to be explored. In particular, we found that the IL‐15 levels were consistently reduced in the CSF after short‐ and long‐term treatment with tofersen. This inflammatory cytokine regulates the long‐lasting activation of T‐cell responses,^29^ and is functionally linked to IL‐2,^30^ whose upregulation could represent a strategy to achieve neuroprotection, as treatments with this protein, alongside brain‐derived neurotrophic factor (also increased by tofersen), are being tested in ALS clinical trials.^31^, ^32^ Thus, the upregulation of inflammatory and glial markers should not necessarily be considered as detrimental.

In addition, the proinflammatory changes of CSF associated with long‐term tofersen treatment may also have influenced the dynamics of supposedly neural markers and contributed to their biphasic behavior. Therefore, UCHL1 levels were effectively reduced by tofersen after 3 months of treatment and remained stable 3 months later, but this reduction unexpectedly vanished at the 12‐month timepoint. Importantly, UCHL1 initially showed a strong correlation with pNfH and NfL, and, as in the case of the neurofilaments, a pronounced UCHL1 increment is observed in the CSF of ALS patients compared with control individuals.^19^, ^33^, ^34^ Hence, an early decrease with similar dynamics in the levels of all 3 aforementioned proteins suggests an acute neuroprotective effect of tofersen. UCHL1 is an enzyme involved in the ubiquitin–proteasome machinery,^35^ which plays an important role in the maintenance of axonal integrity,^36^ but is also upregulated upon inflammation by immune cells.^37^ Thus, and also against the background of persistent reduction of neurofilaments, re‐upregulation of UCHL1 is unlikely to indicate a re‐acceleration of axonal degeneration, but may be associated with the increasingly proinflammatory profile observed in the CSF upon long‐term treatment with tofersen.

A similar principle might also apply to the robust changes of Aβ1–42 and Aβ1–40 observed at 3‐ and 6 months of treatment, which did not persist after 1 year. Consistent with previous studies in sporadic ALS,^38^, ^39^, ^40^, ^41^ the CSF levels of Aβ peptides were increased in the SOD1‐ALS patients of our study, and normalized following treatment with tofersen for 3 months. Aβ isoforms accumulate in neurons, muscle/neuromuscular junctions, and skin of ALS patients, possibly in association with oxidative stress.^42^, ^43^, ^44^, ^45^, ^46^ In addition, APP and BACE1 have been found upregulated at the transcriptional level in SOD1^G93A^ transgenic mice.^47^, ^48^ Of note, our results highlighted a sex‐dependent alteration of Aβ1–40 and Aβ1–42, whose reduction upon tofersen treatment was consistently observed only in male patients. This suggests that the Aβ forms, which might accumulate in patients' biofluids after being released from suffering neurons, could represent a valuable readout to further investigate the early, sex‐specific pathomechanisms recently highlighted by different working groups.^49^, ^50^ Additionally, Aβ mediates inflammatory mechanisms,^51^ and this might explain the re‐elevation of the toxic peptides after prolonged exposure to the ASO. Thus, the (neuro)inflammatory reaction triggered by tofersen might be responsible for time‐dependent alterations in the proteomic profile of patients undergoing therapeutic treatments, and highlight the importance of longitudinally monitoring a panel of heterogeneous biomarkers.

Within this scenario, the altered levels of NPY, which is primarily expressed by interneurons, could represent an endogenous, compensatory (synaptic) mechanism to counteract apoptosis and inflammation.^52^ We indeed found NPY to be increased in CSF of SOD1‐ALS patients in comparison to controls, which is line with previous work describing increased NPY in human post‐mortem ALS samples and revealing that antagonism of the Y1 receptor is sufficient to rescue motor phenotypes in a murine model of ALS.^53^ Notably, the CSF levels of NPY dropped after 3 months of tofersen treatment, indicating an early neuroprotective reaction of this portion of the proteome to the ASO. In contrast, but in line with the dynamics of UCHL1, 12 months after treatment initiation the NPY levels were comparable to those detected at baseline. This might indicate a role for NPY in the pro‐inflammatory response observed at the later stage of treatment,^54^ but also a cell‐specific effect exerted by tofersen on different neuron subtypes. Accordingly, CRH levels in CSF were stably reduced at the treatment timepoints considered in this study and might reflect a reduced degeneration of CRH‐expressing hypothalamic neurons, or an alleviated stress response with reduced synaptic loss.^55^

Furthermore, the well‐established neural damage marker ENO2 (neuron‐specific enolase), the axonal/synaptic markers contactin‐2 (highly expressed in excitatory neurons) and AChE (acetylcholinesterase), as well as the apoptosis marker annexin A5, were decreased after tofersen treatment, whereas GOT1 (glutamate oxaloacetate transaminase 1), which acts as a scavenger of glutamate in the brain, was increased upon ASO therapy. The normalization of GOT1 levels, which are lower in ALS patients than controls, might be interpreted as “metabolic normalization” in neurons, but also in muscle tissue.^56^ Interestingly, combined treatment with recombinant GOT and its cofactor oxaloacetic acid improved motor neuron disease in an ALS rat model.^57^ All these findings suggest that tofersen might exert neuroprotection at the cellular levels already after 3 months of treatment (if not earlier).

In this context, our data support the notion that re‐establishing the excitation/inhibition balance might be an effective therapeutic intervention to influence motor neuron degeneration. The neuropentraxins are candidate markers of synaptic dysfunction in cognitive disorders,^58^ and even if an interaction with SOD1 has not been described yet, they might also reflect a beneficial effect on synaptic homeostasis upon tofersen exposure. The levels of NPTX1, NPTX2, and NPTXR were significantly downregulated within the CSF of ASO‐treated patients, and even correlated with the slower clinical progression observed upon tofersen. Additionally, recent works have highlighted NPTX2 as a key component of TDP‐43 pathology,^59^ and showed that its serum levels correlate with the survival rate of an heterogenous cohort of ALS patients.^60^

In conclusion, our findings indicate that a panel of biomarkers may offer enhanced insight into whether and to what extent a therapeutic agent impacts various aspects of disease pathology, such as neurodegeneration, gliosis, and inflammation, across different stages of treatment. Although analysis of larger cohorts and additional time points is needed to fully understand the effects of tofersen on SOD1‐ALS, our data reveal novel and essential aspects of neuroprotection achieved in treated ALS patients. With further validation and refinement, such a biomarker panel holds promise as a valuable tool for future clinical trials even beyond SOD1‐ALS.

## Author Contributions

D.B. and A.C. contributed to the conception and design of the study; C.S., K.B., F.B., M.W., J.D., J.S., F.S., P.R., M.R., A.G., R.G., M.V., S.P., T.M., T.H., J.G., U.W., P.W., T.H., P.L., J.W., A.H., J.P., K.G., A.K., Z.E., O.P., Z.U., S.W., W.P.R., A.F., R.H., J.H.W., A.L., H.T., and P.O. contributed to the acquisition and analysis of data; C.S., K.B., D.B., and A.C. contributed to drafting the text or preparing the figures.

## Potential Conflict of Interest

Nothing to report.

## Supporting information


**Fig. S1.** Analysis of commonly detectable proteins in serum and CSF. (A) A clear separation of both biofluids is displayed after unbiased hierarchical clustering performed with the 91 proteins commonly detectable. (B) Venn diagram displays 91 commonly detectable proteins in both biofluids. (C) 3D‐representation of PC1, PC2 and PC3 shows clear separation between serum and CSF. (D) Healthy controls show a higher degree of homogeneity within serum and CSF targeted proteome than SOD1‐ALS patients at baseline which was partially normalised upon 3 months of tofersen treatment, accentuated by patient‐centric correlation analysis. Numbers refer to patient numbers as listed in Table S1. (E) Pearson's correlation coefficients reveal increased heterogeneity in ALS‐baseline samples compared to healthy controls (serum vs. CSF), which is normalised upon tofersen treatment. The highest contribution to this underlying heterogeneity rises from serum samples, while CSF shows comparable homogeneity across groups. Data are displayed as mean value ± SD (one‐way ANOVA with post‐hoc Tukey test). Statistical significance was set at *p* < 0.05, exact *p*‐values are displayed.
**Fig. S2.** Comparison between SOD1‐ALS at baseline and healthy controls in serum
Significantly altered proteins in a linear regression model displayed by volcano plot
**Fig. S3.** t‐test analysis between SOD1‐ALS patients at baseline and healthy controls in serum. t‐testing (Welch's t‐test or Mann–Whitney test) revealed significantly (A) downregulated and (B) upregulated proteins. Statistical significance was set at *p* < 0.05, exact *p*‐values are displayed.
**Fig. S4.** Tofersen causes only minor changes to the panel targets in the serum of SOD1‐ALS patients after 3 months of treatment. (A) PC2 and PC3 distinguish SOD1‐ALS from healthy controls (HC) but cannot separate the patients on the basis of ASO treatment, as highlighted in (B) centroid representation of PC2 and PC3. (C) PC‐loadings of PC2 and PC3 combined reveal apolipoprotein E4 and neurofilaments as driving markers from untreated to treated state. Significance was calculated using the linear mixed effect model. Statistical significance was set at *p* < 0.05.
**Fig. S5.** Paired analysis of significantly changed proteins according to linear mixed effect model between 3 months tofersen and baseline in serum. (A) Volcano plot displays significantly altered panel targets between baseline and tofersen‐treated SOD1‐ALS samples. Significantly (B) downregulated and (C) upregulated proteins. Paired *t*‐test was applied. Statistical significance was set at *p* < 0.05, exact *p*‐values are displayed.
**Fig. S6.** Schematic representation of the changes in the serum proteome after 3 months of tofersen treatment. BL = Baseline, Tof = tofersen. Bright blue indicates significant downregulation according to unadjusted *p*‐value, bright red indicates significant upregulation according to unadjusted *p*‐value. *P*‐value was always set as <0.05.
**Fig. S7.** Comparison between the CSF from SOD1‐ALS baseline and healthy controls
Significantly altered proteins in a linear regression model displayed by volcano plot.
**Fig. S8.** t‐test analysis between the CSF from SOD1‐ALS patients at baseline and healthy controls
Comparison between SOD1‐ALS patients and controls (Welch's *t*‐test or Mann–Whitney test) revealed significantly (A) downregulated and (B) upregulated proteins. Statistical significance was set at *p* < 0.05, exact *p*‐values are displayed.
**Fig. S9.** Comparison between CSF of SOD1‐ALS patients at baseline and on tofersen for 3 months. Volcano plot displays significantly altered panel targets between baseline and tofersen‐treated SOD1‐ALS samples. Significance was calculated using the linear mixed effect model. Orange boxes highlight significant markers according to adjusted *p*‐value.
**Fig. S10.** Boxplots of CSF therapy‐responsive biomarkers partly normalised upon 3 months of tofersen treatment
**Fig. S11.** Correlation between Aß‐peptides and neurofilaments in CSF after 6 months of treatment with tofersen. Both (A) Aβ1‐40 and (B) Aβ1‐42 do not significantly correlate with neither pNfH nor NfL between baseline and 6 months.
**Fig. S12.** Comparison between CSF samples from SOD1‐ALS patients after 12 months of tofersen and at baseline. Volcano plot displays significantly altered panel targets between baseline and tofersen‐treated SOD1‐ALS samples at 12 months according to FDR‐adjusted *p*‐value set as <0.05.
**Fig. S13.** Paired analysis of significantly upregulated proteins between 12 months tofersen and baseline condition in CSF reveals mainly inflammatory markers. Significantly altered proteins after 12 months of tofersen treatment.
**Fig. S14.** The patients included in the discovery cohort display a similar response to tofersen and a CSF proteomic profile similar to the patients included in the confirmation group. (A) Except for neurofilaments, all other therapy‐responsive markers selectively analyzed in the discovery‐cohort patients do not display anymore a significant change after 12 months of tofersen treatment. (B) Hierachial clustering of the CSF targeted proteome from the patients of the discovery cohort and the additional ones analyzed after 12 months of treatment did not reveal any clustering between the two sub‐groups of ALS‐SOD1 patients.
**Fig. S15.** Tofersen stabilizes the disease progression in SOD1‐ALS Spaghetti plot showing the stabilisation of the clinical parameters (ALS‐FRS‐R score) upon tofersen treatment.
**Fig. S16.** Correlation between CRH, IL15 and clinical outcome after 12 months of treatment with tofersen
While (A) CRH only slightly correlates with the clinical progress, (B) IL15 does not significantly correlate with the clinical state between baseline and 12 months.

**Table S1.** Supporting information

**Table S2.** Supporting information

## Data Availability

The raw data of the NULISA analysis will be made available upon reasonable request to the corresponding authors.
