# Supplementary material for: Targeted Proteomics upon Treatment with Tofersen Identifies Novel Response Markers for Superoxide Dismutase 1‐Linked Amyotrophic Lateral Sclerosis
Source: Ann Neurol. 2025 Aug 9;98(6):1318–34. doi: 10.1002/ana.70025 (PMC12682945; doi:10.1002/ana.70025)
Supplement: Supplementary file 2 — Table S1. Supporting information [file ANA-98-1318-s001.docx]

| Patient number | Cohort  (months of treatment) | | | Mutation  Zygosity | Sex | Disease duration at baseline (months) | Observed  ALSFRS-R  score | | Expected  ALSFRS-R  score | Delta ALSFRS-R observed - expected  at 12 months  (“Therapy benefit”) | Progression rate (monthly slope of ALSFRS-R) at BL | Progression rate during 12 months of tofersen |
| --- | --- | --- | --- | --- | --- | --- | --- | --- | --- | --- | --- | --- |
|  | 3 m. | 6 m. | 12 m. |  |  |  | BL | 12 m. | 12 m. |  |  |  |
| 1 | X | X | X | p.Asp91Ala (c.272A>C)  Heterozygous | F | 50 | 37 | 35 | 34 | 1 | 0,2 | 0,2 |
| 2 | X | X | X | p.Asp91Ala (c.272A>C)  Heterozygous | M | 39 | 32 | 34 | 27 | 7 | 0,4 | -0,2 |
| 3 | X | X | X | p.Asp91Ala (c.272A>C)  Homozygous | M | 20 | 44 | 43 | 42 | 1 | 0,2 | 0,1 |
| 4 | X | X | X | p.Arg116Gly (c.346C>G)  Heterozygous | F | 6 | 38 | 15 | 17 | -2 | 1,7 | 1,9 |
| 5 | X | X |  | p.Val119_Val12 (c.258-10T>G)  Heterozygous | F | 15 | 34 | - |  |  | 0,9 |  |
| 6 | X | X | X | p.Asp91Val (c.272A>T)  Heterozygous | F | 8 | 37 | 37 | 21 | 16 | 1,4 | 0,0 |
| 7 | X | X | X | p.Leu145Phe (c.435G>T)  Heterozygous | M | 42 | 38 | 36 | 35 | 1 | 0,2 | 0,2 |
| 8 | X | X | X | p.Gly94Cys (c.280G>T)  Heterozygous | M | 14 | 44 | 43 | 41 | 2 | 0,3 | 0,1 |
| 9 | X |  | X | p.Ile114Thr (c.341T>C)  Heterozygous | M | 15 | 41 | 17 | 35 | -18 | 0,5 | 2,0 |
| 10 |  | X | X | p.Leu118Val (c.352C>G)  Heterozygous | M | 225 | 32 | 28 | 31 | -3 | 0,1 | 0,3 |
| 11 |  | X | X | p.Val88Met (c.262G>A)  Heterozygous | F | 124 | 29 | 29 | 27 | 2 | 0,2 | 0,0 |
| 12 |  | X | X | p.Leu145Phe (c.435G>C)  Heterozygous | M | 64 | 18 | 22 | 12 | 10 | 0,5 | -0,3 |
| 13 |  | X | X | p.Arg116Gly (c.346C>G)  Heterozygous | F | 17 | 35 | 32 | 26 | 6 | 0,8 | 0,3 |
| 14 |  | X | X | p.His44Arg (c.131A>G)  Heterozygous | F | 10 | 40 | - |  |  | 0,8 |  |
| 15 |  | X | X | p.Val148Ala (c.446T>C)  Heterozygous | F | 88 | 36 | 32 | 34 | -2 | 0,1 | 0,3 |
| 16 |  | X | X | p.Gly42Asp (c.125G>A)  Heterozygous | F | 29 | 44 | 46 | 42 | 4 | 0,1 | -0,2 |
| 17 |  | X | X | p.His47Arg (c.140A>G)  Heterozygous | F | 17 | 46 | 43 | 45 | -2 | 0,1 | 0,3 |
| 18 |  | X | X | p.Asp91Ala (c.272A>C)  Homozygous | F | 106 | 25 | 26 | 22 | 4 | 0,2 | -0,1 |
| 19 |  | X | X | p.Leu145Phe (c.435G>C)  Heterozygous | F | 19 | 43 | 43 | 40 | 3 | 0,3 | 0,0 |
| 20 |  | X | X | p.Asp91Ala (c.272A>C)  Heterozygous | F | 11 | 41 | 41 | 33 | 8 | 0,7 | 0,0 |
| 21 |  | X | X | p.Val149Gly (c.446T>G)  Heterozygous | M | 6 | 38 | 27 | 18 | 9 | 1,7 | 0,9 |
| 22 |  | X | X | p.His44Arg (c.131A>G)  Heterozygous | F | 47 | 36 | 35 | 33 | 2 | 0,3 | 0,1 |
| 23 |  | X |  | p.His47Arg (c.140A>G)  Heterozygous | M | 26 | 44 | - |  |  | 0,2 |  |
| 24 |  |  | X | p.Asp91Ala (c.272A>C)  Homozygous | M | 40 | 21 | 15 | 13 | 2 | 0,7 | 0,5 |
| 25 |  |  | X | p.Glu101Lys (c.310G>A)  Heterozygous | M | 37 | 42 | 45 | 40 | 5 | 0,2 | -0,3 |
| 26 |  |  | X | p.Asp91Ala (c.272A>C)  Heterozygous | F | 58 | 35 | 38 | 32 | 6 | 0,2 | -0,3 |
| 27 |  |  | X | p.Ala90Val (c.269C>T)  Heterozygous | M | 22 | 41 | 41 | 37 | 4 | 0,3 | 0,0 |
| 28 |  |  | X | p.Ile114Thr (c.341T>C)  Heterozygous | M | 15 | 42 | 40 | 37 | 3 | 0,4 | 0,2 |

**Supplementary Table 1: Clinical information about the cohort of SOD1-ALS patients included in the study**
