# Supplementary material for: Targeted Proteomics upon Treatment with Tofersen Identifies Novel Response Markers for Superoxide Dismutase 1‐Linked Amyotrophic Lateral Sclerosis
Source: Ann Neurol. 2025 Aug 9;98(6):1318–34. doi: 10.1002/ana.70025 (PMC12682945; doi:10.1002/ana.70025)
Supplement: Supplementary file 3 — Table S2. Supporting information [file ANA-98-1318-s002.docx]

**Supplementary Table 2: Clinical information about the control cohort included in the study**

| **Control patient number** | **Diagnosis** | **Sex** | **Age at sampling** |
| --- | --- | --- | --- |
| 1 | Tension headache | male | 63 |
| 2 | Facial palsy | female | 68 |
| 3 | Tension headache | male | 55 |
| 4 | Facial palsy | male | 57 |
| 5 | Tension headache | female | 39 |
| 6 | Sinusitis | female | 36 |
| 7 | Tension headache | female | 51 |
| 8 | Idiopathic intracranial hypertension | male | 61 |
| 9 | Vertigo | female | 57 |
