# Supplementary material for: Targeted Proteomics upon Treatment with Tofersen Identifies Novel Response Markers for Superoxide Dismutase 1‐Linked Amyotrophic Lateral Sclerosis
Source: Ann Neurol. 2025 Aug 9;98(6):1318–34. doi: 10.1002/ana.70025 (PMC12682945; doi:10.1002/ana.70025)
Supplement: Supplementary file 1 — Fig. S1. Analysis of commonly detectable proteins in serum and CSF. (A) A clear separation of both biofluids is displayed after unbiased hierarchical clustering performed with the 91 proteins commonly detectable. (B) Venn diagram displays 91 commonly detectable proteins in both biofluids. (C) 3D‐representation of PC1, PC2 and PC3 shows clear separation between serum and CSF. (D) Healthy controls show a higher degree of homogeneity within serum and CSF targeted proteome than SOD1‐ALS patients at baseline which was partially normalised upon 3 months of tofersen treatment, accentuated by patient‐centric correlation analysis. Numbers refer to patient numbers as listed in Table S1. (E) Pearson's correlation coefficients reveal increased heterogeneity in ALS‐baseline samples compared to healthy controls (serum vs. CSF), which is normalised upon tofersen treatment. The highest contribution to this underlying heterogeneity rises from serum samples, while CSF shows comparable homogeneity across groups. Data are displayed as mean value ± SD (one‐way ANOVA with post‐hoc Tukey test). Statistical significance was set at p < 0.05, exact p‐values are displayed. Fig. S2. Comparison between SOD1‐ALS at baseline and healthy controls in serum Significantly altered proteins in a linear regression model displayed by volcano plot Fig. S3. t‐test analysis between SOD1‐ALS patients at baseline and healthy controls in serum. t‐testing (Welch's t‐test or Mann–Whitney test) revealed significantly (A) downregulated and (B) upregulated proteins. Statistical significance was set at p < 0.05, exact p‐values are displayed. Fig. S4. Tofersen causes only minor changes to the panel targets in the serum of SOD1‐ALS patients after 3 months of treatment. (A) PC2 and PC3 distinguish SOD1‐ALS from healthy controls (HC) but cannot separate the patients on the basis of ASO treatment, as highlighted in (B) centroid representation of PC2 and PC3. (C) PC‐loadings of PC2 and PC3 combined reveal apolipoprotein E [file ANA-98-1318-s003.docx]

**Targeted proteomics upon treatment with tofersen identifies novel response markers for SOD1-linked amyotrophic lateral sclerosis**

Christina Steffke^1,2,3,#^, Karthik Baskar^2,3,#^, Franziska Bachhuber^1,#^, Maximilian Wiesenfarth^1,#^, Johannes Dorst^1^, Joachim Schuster^1,4^, Florian Schöberl^5^, Peter Reilich^5^, Martin Regensburger^6^, Alexander German^6^, René Günther^7,8^, Maximilian Vidovic^7^, Susanne Petri^9^, Thomas Meyer^11^, Tim Hagenacker^12^, Julian Grosskreutz^13^, Ute Weyen^14^, Patrick Weydt^15,16^, Thomas Haarmeier^17^, Paul Lingor^18,19,20^, Joachim Wolf^21^, Andreas Hermann^22,23^, Johannes Prudlo^23,24^, Kornelia Günther^1^, Antje Knehr^1^, Zeynep Elmas^1^, Zeljko Uzelac^1^, Simon Witzel^1^, Wolfgang Philipp Ruf^1,4^, Axel Freischmidt^1^, Ritchie Ho^25,26,27,28^, Albert C. Ludolph^1,4^, Jochen H. Weishaupt^1,10,29^, Hayrettin Tumani^1,4^, Patrick Oeckl^1,4^, David Brenner^1,4,29*^, Alberto Catanese^2,4,30,*^

\# These authors contributed equally to this work.

* These authors contributed equally to this work.

Author affiliations:

1 Department of Neurology, Ulm University, 89081, Ulm, Germany

2 Institute of Anatomy and Cell Biology, 89081, Ulm, Germany

3 International Graduate School, Ulm University, 89081, Ulm, Germany

4 German Center for Neurodegenerative Diseases (DZNE) Ulm, 89081, Ulm, Germany

5 Department of Neurology with Friedrich Baur Institute, LMU University Hospital, LMU Munich, 80336, München, Germany

6 Department of Molecular Neurology, Friedrich-Alexander-Universität Erlangen-Nürnberg (FAU), 91054, Erlangen, Germany

7 Department of Neurology, University Hospital Carl Gustav Carus, Technische Universität Dresden, 01307, Dresden, Germany

8 German Center for Neurodegenerative Diseases (DZNE) Site Dresden, 01307, Dresden, Germany.

9 Department of Neurology, Hannover Medical School, 30625, Hannover, Germany

10 Division for Neurodegenerative Diseases, Neurology Department, Mannheim Center for Translational Medicine, University Medicine Mannheim, Heidelberg University, 68167, Mannheim, Germany

11 Department of Neurology, Center for ALS and Other Motor Neuron Disorders, Charité - Universitätsmedizin Berlin, Corporate Member of Freie Universität Berlin, Humboldt-Universität zu Berlin, and Berlin Institute of Health, 13353, Berlin, Germany

12 Department of Neurology and Center for Translational Neuro and Behavioral Sciences (C-TNBS), University Hospital Essen, 45127, Essen, Germany

13 Precision Neurology of Neuromuscular and Motoneuron Diseases, University of Lübeck, 23538, Lübeck, Germany.

14 Department of Neurology, Ruhr-University Bochum, BG-Kliniken Bergmannsheil, 44789, Bochum, Germany

15 Department for Neuromuscular Diseases, Bonn University, 53127, Bonn, Germany.

16 German Centre for Neurodegenerative Diseases (DZNE) Site Bonn, 53127, Bonn, Germany.

17 Department of Neurology, Helios Klinikum Krefeld, 47805, Krefeld, Germany.

18 Department of Neurology, TUM Klinikum Rechts der Isar, 81675, München, Germany.

19 German Centre for Neurodegenerative Diseases (DZNE) Site Munich, Munich, Germany.

20 Munich Cluster for Systems Neurology (SyNergy), Munich, Germany

21 Department of Neurology, Diako, Brüderklinikum Julia Lanz, Mannheim, Germany

22 Translational Neurodegeneration Section 'Albrecht Kossel', University Medical Center Rostock, 18147, Rostock, Germany.

23 German Center for Neurodegenerative Diseases (DZNE) Rostock/Greifswald, 18147, Rostock, Germany

24 Department of Neurology, University Medical Center Rostock, 18147, Rostock, Germany.

25 Center for Neural Science and Medicine, Cedars-Sinai Medical Center, Los Angeles, CA 90048, USA

26 Board of Governors Regenerative Medicine Institute, Cedars-Sinai Medical Center, Los Angeles, CA 90048, USA

27 Department of Biomedical Sciences, Cedars-Sinai Medical Center, Los Angeles, CA 90048, USA

28 Department of Neurology, Cedars-Sinai Medical Center, Los Angeles, CA 90048, USA

29 Center for Rare Diseases (ZSE) Ulm, Ulm University Hospital Center for Rare Diseases, Ulm, 89081 Ulm, Germany.

30 Institute of Anatomy and Cell Biology, Department of Neuroanatomy, Freiburg University, 79104 Freiburg

Correspondence to:

PD Dr. David Brenner

Department of Neurology, Ulm University,

Oberer Eselsberg 45, 89081 Ulm, Germany

david.brenner@uni-ulm.de

Prof. Dr. Alberto Catanese

Institute of Anatomy and Cell Biology, Department of Neuroanatomy

Freiburg University, Albertstraße 23, 79104 Freiburg, Germany

alberto.catanese@anat.uni-freiburg.de

[alberto.catanese@uni-ulm.de](mailto:alberto.catanese@uni-ulm.de)

**Running title**: Novel therapy-responsive biomarkers in ALS

**Keywords**: amyotrophic lateral sclerosis, tofersen, SOD1, serum, CSF

This file contains 16 supplementary figures
